# Exploring the Factors Influencing Kaohsiung Residents’ Intentions to Choose Age-Friendly Housing

**DOI:** 10.3390/ijerph17217793

**Published:** 2020-10-24

**Authors:** Kun-Kuang Wu, Chun-Chang Lee, Chih-Min Liang, Wen-Chih Yeh, Zheng Yu

**Affiliations:** 1 Department of Real Estate Management, National Pingtung University, Pingtung 90004, Taiwan; wu.caesar@msa.hinet.net (K.-K.W.); 109257503@nccu.edu.tw (Z.Y.); 2 Department of Public Finance and Tax Administration, National Taipei Univeristy of Business, Taipei City 10478, Taiwan; ljimmy.ljimmy@msa.hinet.net; 3 Department of Real Estate Management, HungKuo Delin University of Technology, New Taipei City 23654, Taiwan; wen00126@mail.hdut.edu.tw

**Keywords:** aging in place, multigenerational-multiunit living arrangements, lifetime homes, age-friendly housing, logit model

## Abstract

Taiwan’s declining birthrate has changed the housing market, which should become more consumer-oriented in the future. In particular, age-friendly housing has become a salient housing choice among buyers. Age-friendly housing consists of housing units that are suitable for occupants of any age. There are three concepts underlying such housing: aging in place, multigenerational-multiunit living arrangements, and lifetime homes. This study aimed to examine the factors affecting consumers’ choice of age-friendly housing. The participants were residents of Kaohsiung City, and data analysis was performed using a binary logistic model. The empirical results indicated that adult sons/daughters, residents who currently live in the city center, residents who have a high or medium monthly family income, residents who are currently part of a stem family, residents who desire to live under multigenerational-multiunit living arrangements, residents who desire to be a part of a stem family, and residents who prioritize housing type when house-buying are significantly more likely to choose age-friendly housing. These results can serve as a reference regarding age-friendly housing investments for investors, as well as for house buyers who are deliberating between age-friendly housing and ordinary housing.

## 1. Introduction

Taiwan’s current population structure is reflected in the living arrangements of its citizenry, and is noticeably exhibiting an aging trend, a sub-replacement fertility rate, and a preference for smaller houses. Population aging: In 1991, the World Health Organization (WHO) delineated the indicators of the United Nations Proclamation on Aging. Based on these indicators, a society is an “aging society” when over 7% of its population are aged 65 years and above; an “aged society” if when rate exceeds 14%; and a “super-aged society” when that rate exceeds 20%. According to the statistics of the Ministry of the Interior, Taiwan became an aging society in 1993, and subsequently, an aged society in April 2018 when its elderly population grew to 14.10% of its total population. (Source: https://www.moi.gov.tw/stat/news_detail.aspx?sn=. Last accessed on 2019.8.19.)Sub-replacement fertility: According to the statistics of the Ministry of the Interior, Taiwan’s population decline began in the 1980s when its birth rate decreased annually by 10,000–20,000 births from an initial figure of 400,000 births. (Source: https://zh.wikipedia.org/zh-tw/%E5%B0%91%E5%AD%90%E5%8C%96. Last accessed on 2019.8.19.) This sub-replacement fertility rate has become increasingly pronounced in the past decade, as Taiwan recorded only 181,601 births in 2018. Preference for smaller houses: According to the statistics of the Ministry of the Interior, from 2010 to the first half of 2019, even though Taiwan’s total population had dropped by 457 people, the number of households had increased by 81,295, which indicates a trend of smaller families living in smaller houses while the population is declining. (Source: http://www.kaoarch.org.tw/04–01-01.php. Last accessed on 2019.8.21.)

Forsyth et al. [1] pointed out that given the needs of an aging society, the planning and design of cities, communities, and housing should be centered on age-friendliness to strengthen a community’s functions and create innovative housing products. The physical, social, and service aspects of communities and housing options should be enhanced. To this end, possible measures include diversifying a community’s public infrastructure, providing collective services, establishing age-friendly communities, creating purpose-built specialized housing units, developing intergenerational housing communities, and enriching mobility, care, and communications innovations in communities and housing options. Innovative housing can be examined through two approaches. The first approach is to examine the functions of housing. Before 1980, only the basic functions of a house were typically studied. Between 1980 and 1990, two functions were emphasized: exterior features and quality of living. Between 1990 and 2000, three functions emerged, namely quality and safety, brand images, and building materials. Post-2000, four functions have been emphasized, namely public facilities, property management, green building, and smart building. In light of population aging and sub-replacement fertility, the concept of lifetime homes should be emphasized as well, and it should incorporate accessible design, universal design, and open building.

The second approach relates to housing type, and this approach consists of two phases. In the first phase, consumers emphasize similar housing products, such as mansion apartments, apartment buildings, and townhouses. In the second phase, consumers emphasize different housing products, such as first-time purchases, house swapping, luxury housing, budget-friendly housing, housing for the elderly, housing next to an MRT station, green buildings, and special or environmentally friendly building materials. Both of these phases are house-oriented, while the user (occupant) plays a secondary role. As developments in the housing market shift to a needs-oriented approach, which focuses on the user, a third phase has been introduced-user differentiation. In this phase, housing products are primarily based on users’ needs, and planners should take into account aging societies and sub-replacement fertility rates. In other words, age-friendliness is central when planning such housing options. This phase focuses on two concepts, that is, emphasizing the notion of lifetime homes, which entails accessible design, universal design, and open building, so as to meet the spatial adjustment needs caused by changes in age. Moreover, the interior space and infrastructure of a house should be renewable or customizable according to one’s needs; and overcoming the nuclear family-based housing model by transforming it into a multigenerational-multiunit mixed-housing model. Hwang [2] suggested that the best way to implement long-term care for elderly people is to encourage and enable aging in place as well as multigenerational-multiunit living arrangements. Huang further advocated that age-friendly housing should utilize the following three concepts: aging in place, multigenerational-multiunit living arrangements, and lifetime homes.

In December 2011, the Yungching Charity Foundation conducted a survey on the demand for age-friendly housing. They discovered that as many as 82.6% of Taiwanese expect or strongly expect to live in age-friendly homes in the future. (Source: http://www.ud.org.tw/%e5%8f%96%e5%be%97%e6%a1%88%e4%bb%b6/Last accessed on 2019.8.26.) The use of age-friendly housing in Taiwan was first proposed by Chen [3]. The author’s study showed that new housing types should incorporate new family structures and multigenerational-multiunit living arrangements, so as to strengthen the bond between different generations in a family and promote accessible and timely care services while maintaining affection between caregivers and care recipients. Age-friendly housing should be designed as lifelong housing, in which not only the special needs of the disabled and the elderly are taken into account, but also differences among generations, genders, and urban/rural areas. Such housing should be accessible and disabled-friendly and create secure, convenient, and comfortable living environments. To date, the age-friendly housing model proposed in the present study remains relatively unfamiliar to consumers. Our model was built on the three components of age-friendly housing (i.e., aging in place, multigenerational-multiunit living arrangements, and lifetime homes), as well as the three design concepts of lifetime housing (i.e., accessible design, universal design, and open building). The participants in this questionnaire-based study consisted of Kaohsiung residents. The objectives of this study were to explore the influences of the participants’ socioeconomic characteristics, current housing product attributes, and desired housing product attributes on their choice of age-friendly housing and ordinary housing. Binary logistic regression was used to analyze the factors influencing consumers’ choice of age-friendly housing and ordinary housing.

## 2. Literature Review

Age-friendly housing is an integrated concept that consists of three components: aging in place, multigenerational-multiunit living arrangements, and lifetime homes. In reality, aging in place includes aging at home and aging in the community. Aging in place views aging as a normal part of human growth. Therefore, the aging process should occur naturally for elderly people in the settings they live in. Elderly people should not be forced to leave the settings that they have lived in for decades on the basis of ill health and be cared for in nursing institutions. The notion of aging naturally is based on the quality of life of elderly people. Multigenerational-multiunit living emphasizes the needs of parents. In Chinese societies, elderly people feel proud when they are being cared for by their own sons/daughters. Meanwhile, through this caregiving approach, they are able to interact with their sons/daughters and provide and receive support. In terms of the needs of sons/daughters, filial piety, resource assistance, and mutual relationships act as links that strengthen the bond between different generations. The concept of lifetime homes revolves around aging in place; and stresses that people can live independently and autonomously in their own homes throughout their lives instead of staying in a nursing institution. Even though their declining bodily functions create various inconveniences, these problems can be overcome by re-designing and renovating their living environments. In other words, the environment of a lifetime home should be able meet the needs of occupants at different stages of life. Non-occupants, on the other hand, can meet their needs by moving to new houses and readapting themselves. Various studies have discussed the functions and means of promoting aging in place, but few have examined the specific issue of lifetime homes. For instance, Szanton et al. [4] examined whether a family care plan could reduce diseases and promote aging in place. Their results showed that the plan could potentially enhance the elderly’s ability to age in place. Kim et al. [5] and Wang et al. [6] defined aging in place as an elderly person’s ability to maintain an independent and high quality of life in their home or in their community, and proposed that this could be realized by digital technology. Ahn et al. [7] stated that perceptions of well-being could be a driver or result of aging in place.

Sanguinetti [8] administered a questionnaire to investigate the satisfaction of elderly people aged 60 years or older with living in 12 intergenerational housing communities in North Carolina. The results showed that a multigenerational-multiunit living arrangement was healthier than living with people in the same age group. In light of the impending aging of American society, Scheidt [9] proposed several concepts pertaining to age-friendly housing concepts such as lifetime homes, age-friendly lifelong communities, and multigenerational-multiunit living arrangements, so as to overcome the problems of living arrangements for the elderly. Xie et al. [10] examined the development of age-friendly retirement communities in China. The authors consolidated studies and data pertaining to representative and well-established age-friendly retirement communities in China, identified the functional and spatial needs of elderly groups, presented a model for the development and arrangement of age-friendly retirement communities in China, and elucidated points that should be taken into account during the early planning phase of such retirement communities. Chen [11] examined the means of incorporating smart devices, software, and networking functions into the lives of elderly people, so as to build comfortable, friendly, secure, energy-saving, and convenient age-friendly and smart housing.

Due to the lack of age-friendly housing studies, this study aimed to examine the factors affecting consumers’ choice of age-friendly housing. We reviewed studies related to the factors that influence consumers’ choice of different housing types. This field of research is often included as a part of research on consumers’ purchase decisions and housing choices. Clark and Onaka [12] showed that four variables, monthly family income, desired living location, desired family pattern, and desired area of purchased house, have significant expected impacts on the housing types chosen. Consumers with a higher monthly family income, a higher number of desired housing occupants, and a higher desired area of purchased house have a higher probability of prioritizing certain housing types compared to housing locations when buying a house. Bhat [13] demonstrated that in addition to factors pertaining to the interior and exterior of a house, the socioeconomic characteristics of a consumer also influence their housing choices. Bonnet [14] indicated that family monthly income, desired housing location, crime rate in the desired housing location, public infrastructure in the desired housing location, and property taxes have significant and different impacts on consumers’ choice probability with regard to desired housing type and housing location. In his study, Coulter [15] found that occupation, monthly family income, education level, current home ownership, and current housing prices in a location have significant impacts on the choice probability of desired housing types and locations. More specifically, white-collar parents who work as managers, families with a high family income, parents who currently own the family house, and families who are currently living in areas with high-priced homes have a higher degree of freedom in terms of housing choices, as well as a higher probability of parent-child co-residence.

Interestingly, Hsieh and Tsai [16] demonstrated that current home ownership, transportation, economic climate, and sense of place have significant and varying impacts on housing choices and the choice probabilities of housing locations. By taking the Central Taiwan Science Park as an example, the authors showed that when the younger cohort have a stronger positive influence on the local transportation and economic climate, they would be more willing to stay put in their current housing location, develop a deeper sense of place, and be less likely to move out. The key variable for the older cohort was also sense of place, as a stronger sense of place increased the likelihood of them settling down permanently in the area. Moreover, people who have full ownership of their houses are less likely to move out.

## 3. Research Method

### 3.1. Logistic Regression Model

In this study, age-friendly housing refers to houses with a living environment that is suitable for an occupant to live in throughout their entire life (from childhood to late adulthood). It also serves as a space where parents and sons/daughters can co-reside. Sons/daughters have more needs related to leisure and entertainment as well as information and media; parents have more needs related to elderly care as well as social interactions. Based on housing design concepts, this study incorporated four types of occupants’ needs-leisure and entertainment, information and media, elderly care, and social interactions-into the construction of a comprehensive framework on the planning and design of age-friendly housing. This approaches allows for multigenerational-multiunit living arrangements, which are a new form of living arrangement. House planners play the most salient role in the process of building age-friendly housing, and they should pay attention to the procedural requirements in house planning.

The design concepts of age-friendly housing should be procedural and are as follows: (1) With regard to vertical circulation, elevators are required so that wheelchair-bound occupants can move around with their attendants and reach public areas. (2) In terms of horizontal circulation, thresholds should be removed from the main entrance, bathrooms, kitchen, and rooms of a housing unit. Indoor and outdoor pathways should be wide enough for ambulatory devices to pass through. (3) In terms of external circulation, pathways should be designed and linked such that a person can easily travel from the entrance of their house, to the elevator of their floor, to the main entrance of their housing building, and finally to a point for further transportation. (4) The dimensions of facilities and spaces should be disabled-friendly and comply with accessible design requirements. (5) During the planning process, spaces should be reserved in housing buildings according to the principles of open building such that modifications can be made flexibly.

The dependent variable (BCHOICETYPE) in this study was a binary variable in which the consumers either chose age-friendly housing (set as 1) or ordinary housing (set as 0). We employed a binary logistic model to analyze which factors influenced the respondents’ (consumers’) choice of age-friendly housing. The model was set up as shown below:

(1) $$BCHOICETYPE_{i}=\frac{1}{1+e^{-LOR_{i}}}\;+u_{i}$$

(2) $${LOR}_{i}=\log(\frac{p_{i}}{1-p_{i}})=\beta_{0}+\beta_{1}{GEN}_{i}+\beta_{2}{SEX}_{i}+\beta_{3}{CLOC}_{i}+\beta_{4}{INCOME1}_{i}+\beta_{5}{INCOME2}_{i}\;+\beta_{6}{EDUH}_{i}+\beta_{7}{EDUM}_{i}+\beta_{8}{COWNSHIP1}_{i}+\beta_{9}{COWNSHIP2}_{i}+\beta_{10}{CTYPE}_{i}\;+\beta_{11}{HLOC}_{i}+\beta_{12}{HLIVETIPE1}_{i}+\beta_{13}{HLIVETIPE2}_{i}+\beta_{14}{HFAMTYPE}_{i}+\beta_{15}{BCHOICE}_{i}\;+\beta_{16}{HUPH}_{i}+\beta_{17}{HUPM}_{i}$$

in which i represents a respondent. $p_{i}$ represents the respondent’s probability of choosing age-friendly housing. $1-p_{i}$ represents the respondent’s probability of choosing ordinary housing. $LOR_{i}$ is the logarithm of the odds of choosing age-friendly housing. $u_{i}$ is the error term.

The explanatory variables in this study included socioeconomic characteristics, current housing attributes, and desired housing attributes. The socioeconomic characteristics consisted of the house buyers’ generation (GEN), gender (SEX), current living location (CLOC), monthly family income (INCOME1, INCOME2), and education level (EDUH, EDUM). The current housing attributes consisted of the current home ownership (COWNSHIP1, COWNSHIP2) and current family type (CTYPE). The desired housing attributes consisted of the desired housing location (HLOC), desired living arrangement (HLIVETYPE1, HLIVETYPE2), desired family type (HFAMTYPE), house-buying priorities (BCHOICE), and desired housing price (HUPH, HUPM). $\beta_{0}$ is the intercept and $\beta_{1}\sim \beta_{17}$ is the coefficient of an independent variable. The definitions of the variables are listed in Table 1.

### 3.2. Description of Variable Settings

#### 3.2.1. Dependent Variable

This study first divided housing products into age-friendly housing and ordinary housing. The dependent variable (BCHOICETYPE) was a binary variable in which respondents who chose age-friendly housing were set as 1 while the others choosing ordinary housing were set as 0.

#### 3.2.2. Independent Variables

Since age-friendly housing options are relatively new, there is a dearth of research on the factors influencing house buyers’ choice of age-friendly housing. The independent variables in this study included socioeconomic characteristics, current housing attributes, and desired housing attributes. The socioeconomic characteristics consisted of a consumer’s generation, gender, current housing location, monthly family income, and education level. The current housing attributes consisted of current home ownership and current family type. Lastly, the desired housing attributes consisted of desired housing location, desired housing type, desired family type, house-buying priorities, and desired housing price (see Table 1).

(1) Socioeconomic characteristics

Wang [17] pointed out that a multigenerational-multiunit living arrangement is a familial living arrangement in which family members of different generations live in separate housing units. It is described as a “modified big family,” and people who choose this living arrangement are influenced by parent-child co-residence. Yi and Lin [18] conducted a study on adult sons/daughters and derived five types of the participants’ desired living arrangements with their parents: normative, detached, tight-knit, sociable, and intimate but distant. The normative type was the most prominent according to a logit empirical analysis. This relational type could be a result of the interaction between personal resources and traditional filial piety norms, as parents strongly depend on their sons/daughters to provide care for them, and thus zealously instill traditional filial norms in their sons/daughters. The adult sons/daughters on the other hand, perceive that their parents are able to provide them with socioeconomic resources, and hence desire to co-reside with their parents. For the generation (GEN) variable, this study utilized an age of 65 years and above as a reference group (In this study, the age of 65 served as a cutoff value, and elderly people aged above 65 years served as a reference group. More specifically, old age is defined as follows: 1. The WHO defines an elderly person as one over 65 years of age. (Source:https://www.happyold.net/3750439662260632345032681.html); 2. According to Taiwan’s Senior Citizens Welfare Act Article 2, elders are people who are aged above 65 years old; 3. According to the National Development Council, Executive Yuan, old age is defined as being over 65 years old. (Source:https://pop-proj.ndc.gov.tw/chart.aspx?c=10&uid=66&pid=60 Last accessed on 2020.04.18)), and older adults aged above 66 years were set as 1 while younger adults under 66 years of age were set as 0. In contrast, parents had a higher preference to co-reside with their sons/daughters or live in the same community with them. Wang [19] performed a cross-analysis of co-residence tendencies and living arrangements, and found that parents were more inclined to co-reside with their sons/daughters than their sons/daughters were to co-reside with their parents.

Yi and Lin [18] pointed out that increasing socioeconomic resources and declining filial norms have provided more opportunities for adult sons/daughters to live on their own. We surmise that the coefficient of the generation variable is a positive value. With regard to gender, Huang [20] indicated that adult sons were more inclined to co-reside with their parents than adult daughters. Wang [19] pointed out that according to Taiwanese societal customs, married men are obliged to be filial, while married women have to live with their in-laws. Shih [21] suggested that due to parenting cultures, married Taiwanese women often have to deal with the differences between their cultural perceptions and parenting methods and those of their in-laws, whereas their husbands do not as their wives are married into their families. Therefore, males have a higher propensity toward choosing age-friendly housing than do females. In this study, gender was designed as a dummy variable, with male being set as 1 while female was set as 0. We surmise that the coefficient of the gender variable has a positive value.

With regard to current living location, Huang [22] indicated that house buyers were more willing to choose houses in city centers that offer better housing prices, convenience, and livability than other areas. Naess et al. [23] indicated that the housing choices of residents in the city center were based on high quality facilities and the shortest commuting distance. Age-friendly housing units also provide more facilities than ordinary housing units, and public infrastructure in urban areas is generally of a better quality than that in suburban areas. In this study, current living location was designed as a dummy variable, in which city centers were set as 1 and suburban areas were set as 0. We surmise that the coefficient of the current living location variable has a positive value.

A higher monthly family income affects house buyers’ housing choices. Hsiang [24] mentioned that house buyers in Kaohsiung City with a monthly family income exceeding NT$200,000 place greater emphasis on the features of a house. Coulter [15] pointed out that parents with a higher average monthly income have a higher propensity toward owning their own home, while due to their financial abilities, the sons/daughters of such parents are more likely to co-reside with their parents. Since age-friendly housing options are costlier than ordinary housing options, buyers with higher incomes are more likely to choose age-friendly housing. We divided monthly family income into low (less than NT$60,000), middle (between NT$60,000 and NT$120,000), and high (over NT$120,000) categories, of which the low income category served as a reference, with two dummy variables (INCOME1 and INCOME2) being designed. For the former dummy variable, high income buyers were set as 1, while others were set as 0; for the latter, middle income buyers were set as 1, while others were set as 0. We surmise that the coefficient of the income variable has a positive value. 

Lastly, with regard to education level, Tseng et al. [25] showed that parents with higher education levels are more likely to live in the same neighborhood as their adult sons/daughters. Coulter [15] indicated that parents with higher education levels have higher levels of intergenerational interactions. In this study, education level was divided into low, medium, and high levels, of which the low education level served as the reference group, with two dummy variables (EDUH and EDUM) being designed. For the former dummy variable, high education level was set as 1, while the medium and low education levels were set as 0. For the latter, medium education level was set as 1, while the high and low education levels were set as 0. We surmise that the coefficient of the education level variable has a positive value.

(2) Current housing attributes

With regard to current housing attributes, Tseng et al. [25] demonstrated that Taiwanese parents who can better accept living in the same neighborhood as their sons/daughters enjoy better mental and physical health. In addition, resource exchanges are more likely to occur with married sons/daughters, that is, the parents may own the house. Kuo [26] studied the influence of the characteristics of elderly parents and their sons/daughters, as well as the sons/daughters’ concept of filial piety, on the living arrangements of elderly parents. The results showed that due to living arrangements and high dependency, parents (or spouses) who currently own a house have a higher propensity to co-reside with their sons/daughters. On the other hand, due to filial norms, sons/daughters are willing to co-reside with their parents even if they own a house. Lin and Peng [27] highlighted that in the six special municipalities of Taiwan, due to the difficulty of buying their own homes, more adult sons/daughters are choosing to co-reside in their parents’ house. This indicates that the wealth of parents has gradually become a determinant of their sons/daughters’ living arrangements. If parents own a house, then their sons/daughters may co-reside with them; if parents own two houses in the same community, their sons/daughters may choose to live in the other house. In this study, there were three types of current home ownership: consumers’ (or their spouse’s) sole ownership; joint ownership between parents and sons/daughters; and tenancy and others, in which tenancy and others served as a reference group, with two dummy variables (COWNSHIP1 and COWNSHIP2) being designed. For the former dummy variable, sole ownership was set as 1, while joint ownership and tenancy and others were set as 0. For the latter, joint ownership was set as 1, while sole ownership and tenancy and others were set as 0. We surmise that the coefficients of the COWNSHIP1 and COWNSHIP2 variables have positive values.

With regard to family type, Chang and Chang [28] pointed out that family values are gradually being replaced by the exchange theory. Even though at present, the nuclear family type remains the dominant family type, according to the exchange theory, interests, prestige-related, and labor-related factors will cause nuclear families to become stem families. According to the 1998 “Summarized Report on the Survey Results of Social Development Trends in Taiwan,” which investigated family types, 51.15% of families consisted of a couple with unmarried sons/daughters; 8.34% of families consisted of a childless couple or a couple whose sons/daughters had moved out; and both of these types of families are typical nuclear families, which jointly accounted for 59.49% of all families, with an average of 3.91 members per family. Furthermore, based on the studies by Lin [29] and Chien and Yi [30], the number of people in a household was taken as a general rule without taking into account special cases. The data were combined adequately, and families were divided into nuclear families and stem families based on the number of members. In this study, family type was determined by the number of family members. A family with one to four members was regarded as a nuclear family, while a family with five or more members was regarded as a stem family.

Forsyth et al. [1] highlighted that there are many places in the world where elderly people want to age in their own homes in ways that are “appropriate for their age.” Even though these elderly people yearn for new types of houses that meet their self-care, mobility, and household self-management needs, they still prefer to live in current or new houses with other generations. Chao [31] argued that elderly ethnic Chinese people have a stronger sense of independence but are still highly dependent on others. They feel proud to be cared for by the sons/daughters they raised, and hence arrange for their sons/daughters to live with them in the same neighborhood. In this study, nuclear families served as a reference, and a dummy variable was designed. Stem families consisting of five members or more were set as 1, while nuclear families consisting of one to four members were set as 0. We surmise that the coefficient of the family type variable has a positive value.

(3) Desired housing attributes

With respect to desired living location, Lee [32] reported that elderly people prioritize access to medical treatment in their living environments, and thus have a higher propensity toward choosing to live in developed urban areas. Hu [33] administered a questionnaire to residents in Hsinchu City’s East, North, and Siangshan districts and employed a binary logit model for data analysis. The results of that study indicated that ease of transportation, cost of commuting, public infrastructure and services, and urban safety system were the main factors influencing younger adult residents’ choice of living location. Huang’s [22] study showed that convenience influenced residents’ choice of age-friendly housing, as the dining options, commercial districts, leisure environments, medical institutions, cultural and educational institutions, and sports facilities around a community realize the residents’ needs for a convenient living location, and such facilities are more ubiquitous in city centers compared to suburban areas. In this study, for the desired living location variable, suburban areas served as the reference group, and a dummy variable (HLOC) was designed, for which city centers were set as 1, and suburban areas were set as 0. We surmise that the coefficient of the desired living location variable has a positive value. 

With respect to desired living arrangement, Wang’s [17] study showed that parents and sons/daughters who live in the same neighborhood enjoy more benefits than those who co-reside or live alone. This new living arrangement reflects the change in the population structure of today’s society. This study divided living arrangement into three types based on distance: parent-child co-residence, living in the same community with each other, and living as far apart as possible. The latter served as the reference group, and two dummy variables (HLIVETYPE1 and HLIVETYPE2) were designed. For the former dummy variable, parent-child co-residence was set as 1, while other living arrangements were set as 0. For the latter, parents and sons/daughters living in the same community was set as 1, while other living arrangements were set as 0. We surmise that the coefficients of the desired living arrangement variables have positive values.

With regard to house-buying priorities, house buyers prioritize two factors: housing type (age-friendly housing and ordinary housing) and housing location (city center or suburban area). Lein [34] indicated that with regard to consumers’ house-buying preferences, they are more inclined toward prioritizing the type of a house compared to its location. In this study, and a dummy variable (BCHOICE) was designed with prioritizing housing location serving as the reference group. Consumers who prioritized housing type were set as 1, while consumers who prioritized housing location were set as 0. We surmise that the coefficient of the house-buying priorities variable has a positive value.

Two desired family types were established, stem families (five members or more) and nuclear families (one to four members), of which nuclear families served as a reference group, with a dummy variable (HFAMTYPE) being designed. Consumers who desired a stem family were set as 1, while consumers who desired a nuclear family were set as 0. We surmise that the coefficient of the desired family type variable has a positive value.

With regard to housing unit prices, this study emphasized locational factors when examining consumers’ desired housing attributes. Housing location has a direct and positive effect on housing unit prices, and the total price is the product of a house’s unit price and its area. The unit price is a single independent variable, and housing area must be taken account when investigating the total housing price, or else errors may arise during model prediction. Tefera [35] indicated that consumers primarily emphasize unit price sensitivity in their housing choices and that that factor directly influences their choice of housing location and type. This study employed desired housing unit price as an independent variable. There were three types of desired housing unit prices: high (more than NT$180,000), middle (between NT$140,000 and NT$180,000), and lower (NT$140,000), among which lower-priced housing served as a reference group, with two dummy variables (HUPH, HUPM) being designed. For the former dummy variable, high-priced housing was set as 1, while middle- and lower-priced housing were set as 0. For the latter, middle-priced housing was set as 1, while high- and lower-priced housing were set as 0. Assuming that consumers have a lower likelihood of choosing high-priced age-friendly housing, we surmise that the coefficient of the desired housing unit price variable has a negative value.

How a household decides to pay for their housing does indeed entail discussions between family members, but such observations are difficult to capture through empirical methods. In general, the head of household’s background data serves as a factor that influences such decisions. As age-friendly housing is a relatively new concept, there is a lack of theoretical models that could provide relevant insights. Hence, this study took an exploratory approach to identify the factors that influence or do not influence consumers’ choice of age-friendly housing, such as the differences among generations.

(4) Questionnaire design

The contents of the questionnaire included: 1. The respondents’ socioeconomic characteristics, such as age (generation), gender, current living location, monthly family income, and education level; 2. Current housing attributes, such as current house ownership and current number of family members living in the house (family type); 3. Desired housing attributes, such as desired living location (city center or suburban area), desired housing type (age-friendly or ordinary housing), desired living arrangement, desired number of family members living in the house (family type), house-buying priorities, and desired housing unit price. Details of the questionnaire are available upon request. (Appendix A)

## 4. Data Description and Sample Statistics

### 4.1. Data Collection

The participants of this study consisted of people aged above the legal age of 18 living in multi-family housing units in Kaohsiung City. Kaohsiung City has a total of 38 administrative districts since merging with the former Kaohsiung County. This study selected districts with better convenience of living where the average price of a new house exceeds NTS180,000 per *ping* (1 ping is equal to 35.58 feet.). There are seven such districts- Sinsing, Cianjin, Lingya, Sanmin, Gushan, Tsoying, and Fongshan, and are collectively referred to as the “city center.” Eight other neighboring districts- Yancheng, Cianjhen, Siaogang, Nanzih, Ciaotou, Renwu, Niaosong, and Daliao, are collectively referred to as the “suburban area.”

This study employed convenience sampling to collect data from 15 administrative districts in Kaohsiung City, in which seven are located in the city center and eight are located in the suburban area. Kaohsiung City consists of 38 administrative districts. We selected 15 districts because seven of the districts form the city center, where a housing unit in a residential building costs more than NT$180,000 per ping; while eight of the districts form the suburban area, where a housing unit in a residential building costs between NT$160,000 to NT$180,000 per ping. Meanwhile, the main housing product in the 23 other districts, which are regarded as the outskirts of Kaohsiung City, was not residential buildings; and even if there are such buildings in these districts, a unit would cost less than NT$160,000 per ping. Moreover, in convenience sampling, the sample size must be taken into account as it affects the accuracy of the estimated results. We assumed a tolerable error of 0.05 at a 10% level of significance, that is, the required sample size is 271 responses at a 90% confidence level. A total of 20 different households from different housing communities were selected from each of the 15 districts. A total of 20 different households from different housing communities were selected from each of the 15 districts. A total of 210 questionnaires were administered to the seven districts in the city center (30 questionnaires for each district). A total of 240 questionnaires were administered to the eight districts in the suburban area (30 questionnaires for each district). A total of 450 questionnaires were administered in this study. Convenience sampling was employed; households who declined to participate were excluded, and another household was selected instead. Survey takers were hired and trained rigorously. Each returned questionnaire was checked for uncompleted items. In such cases, the participant was contacted and asked to respond to the item. 450 questionnaires were administered, all of which were returned. After removing 150 invalid responses, there were 300 valid responses, which indicated an effective recovery rate of 66.7%. A total of 140 questionnaires were administered to the seven districts in the city center. A total of 160 questionnaires were administered to the eight districts in the suburban area. The questionnaire was administered from November 30, 2019 to December 30, 2019.

### 4.2. Descriptive Statistics of Samples

A total of 300 questionnaires were administered in this study, and all responses were valid. The results of the descriptive statistics analysis of the responses are shown in Table 2.

#### 4.2.1. Socioeconomic Characteristics of Respondents 

The youngest and oldest respondents were 20 and 82 years of age, respectively. The mean age of all respondents was 45.52 years. In terms of generational grouping, parents (aged 66 years and above) accounted for 5.7% of respondents, while sons/daughters (aged 66 years and below) accounted for 94.3%. According to the cutoff point of 66 years, a large majority of the responses was based on the perspectives of adult sons/daughters. In terms of gender, males accounted for 47.0% of respondents and females accounted for 53.0%. In terms of current living location, 46.7% were living in the city center and 53.3% were living in the suburban area. Respondents with a low (less than NT$60,000) and medium (NT$61,000 to NT$120,000) monthly family income each accounted for 41.3%, while only 17.4% of respondents had a high (more than NT$121,000) income. Most (49.0%) of the respondents had a medium education level (senior high school and junior college); followed by those with a high education level (university and above), who accounted for 42.7%; while only 8.3% had a low education level (elementary school and below).

#### 4.2.2. Current Housing Attributes

A majority (43%) of the respondents (or their spouses) currently owned their own homes, followed by joint ownership between parents and sons/daughters, which accounted for 37%, while tenancy and others only accounted for 20% of the sample. In other words, non-sole ownership accounted for 57% of the sample. Nuclear and stem families accounted for 68.3% and 31.7% of the sample, respectively.

### 4.3. Desired Housing Attributes

In terms of desired housing type, 50.3% of the respondents desired age-friendly housing while 49.7% of them desired ordinary housing. In terms of desired living location, 73.7% of the respondents desired to live in the city center and 26.3% desired to live in the suburban area. In terms of desired living arrangement, 34.7% of the respondents stated that they desired to co-reside with their sons/daughters/parents, followed by 33.0% who desired to live in the same area, and then 27.7% who desired to live in the same neighborhood, while only 4.7% desired to live as far apart as possible. In terms of desired family type, 73.7% of the respondents desired to be part of a nuclear family while 26.3% desired to be part of a stem family. In terms of house-buying priorities, 59.0% of the respondents prioritized housing location (city center or suburban area) while 41.0% prioritized housing type (age-friendly or ordinary housing). In terms of desired housing unit price, 38.7% of the respondents desired lower-priced housing (less than NT$140,000 per *ping*), followed by 35.3% who desired middle-priced housing (NT$141,000 to NT$180,000 per *ping*), and 26% who desired high-priced housing (more than NT$181,000 per *ping*).

## 5. Empirical Analysis Results

The empirical results are presented in Table 3. The likelihood-ratio (LR) chi-square statistic was 64.10, with a *p*-value of 0.001 (*p* < 0.05), which indicated that this study’s binary logit model of age-friendly housing was statistically significant.

Firstly, in terms of the respondents’ socioeconomic characteristics, the coefficient of the generation variable was −1.623, and the odds ratio was 0.197 at a 5% level of significance. This suggests that the logarithm of the odds of parents choosing age-friendly housing was 1.623 less than that of their sons/daughters. In other words, the odds of parents choosing age-friendly housing was only 0.197 times that of their sons/daughters. Therefore, this result was not in line with what we had surmised. Hsieh and Tsai [16] suggested that the key factor affecting the housing choices of elderly people is sense of place; whereas quality of living was the key factor affecting younger generations. If age-friendly housing becomes a new housing product that offers a better quality of living, then the younger generation would show a higher acceptance. Li and Hung [36] studied the living arrangements of the younger Taiwanese cohort and showed that due to their parents’ resources, younger Taiwanese are more likely to co-reside with their parents or live near them. This finding contrasts with the Western tradition of moving out when one is of age. This effect had a marginal relation with age and gender, and correlated positively with the financial ability and housing choices of the younger generation.

Gender had an estimated coefficient of −0.15 and an odds ratio of 0.860, which did not attain a level of significance. This suggests that females have a higher probability of choosing age-friendly housing compared to males. Even though the value of the coefficient differed from our expectations, the effect was not significant. Chang [37] analyzed domestic migration raw data between 1992 and 2007 by means of conditional logit modeling. The results indicated that due to their jobs and income, Taiwanese females have an absolute autonomy and control over their housing choices. They preferred housings that are idyllic and are located in places that offer a high quality of living. Furthermore, Liu et al. [38] administered a questionnaire to Kaohsiung residents and indicated that rigorous safety monitoring and holistic elderly care influenced consumers’ purchases of high-quality housing, and these two factors are salient design concepts for age-friendly housing. They also found that age-friendly housing met the housing needs of females.

Current living location had an estimated coefficient of 0.866 and an odds ratio of 2.377 at a 1% level of significance. This suggests that the odds of respondents who live in the city center choosing age-friendly housing was 2.377 higher than those living in the suburban area. Hsu [39] conducted a questionnaire survey on residents in Taipei City’s metropolitan area and revealed that convenient living functions, diverse commercial spaces, comprehensive neighborhood services and management, and robust household finances were four aspects that have positive effects on the residents’ satisfaction with their quality of life. Huang [22] indicated that residents living in the city center where housing prices are better, living is more convenient, and livability is more enhanced are more willing to buy age-friendly housing compared to residents living in other areas. The empirical results of the present study correspond to what we had surmised. 

The estimated coefficients of high and middle income were 1.109 and 0.538, respectively, and the odds ratios were 3.032 and 1.712, respectively, and attained a 5% and 10% level of significance, respectively. This suggests that the odds of high- and middle-income respondents choosing age-friendly housing were 3.032 and 1.712 higher, respectively, than that of low-income respondents. Chang et al. [40] reported that parents who have high income and contribute to household income as well as sons/daughters with high social status and income have a higher propensity to choose age-friendly housing, with the latter also being influenced by social prestige. Chen and Lin [41] demonstrated that houses are purchased for self-use or for investment purposes. Buyers with higher income regard housing as a symbol of their wealth, and have high demands for the number, quality, and tenancy of their houses. These buyers also have a higher propensity to choose ideal living locations and private housing. The empirical results support what we had surmised.

The estimated coefficients of high and medium education level were −0.305 and −0.703, respectively, and the odds ratios were 0.737 and 0.495, respectively; none of them were statistically significant. This suggests that respondents’ education level did not lead to any significant differences on their choice of age-friendly or ordinary housing. Tseng et al. [25] showed that parents with a higher education level are more likely to live in the same neighborhood as their adult sons/daughters. According to Yi and Chang [42], the family structure in Taiwan has gradually reverted to the patriarchal living arrangement (three generations living in the patriarchal home), which resulted from an increasingly educated Taiwanese population, elevating human capital for males and females, increased household income from two working parents, and adult sons/daughters who lack the time for household chores. However, the empirical results do not support what we had surmised. 

Secondly, in terms of the respondents’ current housing attribute of current house ownership, the coefficient of sole ownership was 0.256 and the odds ratio was 1.292, but did not attain a level of significance. Joint ownership had an estimated coefficient of −0.237 and an odds ratio of 0.789, which did not attain a level of significance. This suggests that respondents’ joint ownership and tenancy did not lead to any significant differences in their choice probability of age-friendly or ordinary housing. Chen [43] suggested that the based on traditional ethnic Chinese norms, consumer who turn their house ownership from tenancy to sole ownership indicates a better housing quality. However, in practice, a change in ownership does not represent enhanced housing quality and can instead lead to poorer housing environments, quality of living, and higher housing debt. The empirical results suggest that current house ownership did not lead to any significant differences in the respondents’ choice probability of age-friendly or ordinary housing. 

Current family type had an estimated coefficient of 0.589 and an odds ratio of 1.802 at a 10% level of significance. This indicates that the odds of stem families choosing age-friendly housing were 1.802 higher than that of nuclear families. Chang et al. [40] elucidated that parents in stem families have higher social and economic statuses, and are the main source of income for a household. Sons/daughters who have relied economically on their parents for extended periods also have a higher likelihood of maintaining a stem family. Chang and Chang [28] pointed out that family values are gradually being replaced by the exchange theory, that is, parents care for their grandsons/granddaughters, do housework, or manage the household’s socioeconomic resources in exchange for being cared for by their sons/daughters. Even though at present, the nuclear family type remains the dominant family type, but interests, prestige-related, and labor-related factors would cause nuclear families to become stem families. Nuclear families are small families with fewer members, and in many of these families, parents co-reside with their sons/daughters. There is an overt gap between these families’ choice of age-friendly housing, which cater for multigenerational-multiunit living arrangements. The empirical results support what we had surmised.

Thirdly, the estimated coefficient of desired living location was −0.509, which had a different symbol that expected, and an odds ratio of 0.601, which did not attain a level of significance. This suggests that living in the city center or suburban area did not lead to any significant differences on the respondents’ choice probability of age-friendly or ordinary housing. Many areas in suburban Kaohsiung have been rezoned for urban development. Kim and Horner [44] highlighted that house owners who currently live in slums, declining areas, and overpopulated areas have a higher likelihood to move to urban renewal zones, urban redevelopment zones, and areas where neighboring roads are being constructed or broadened. Yet, the empirical results did not support what we had surmised.

In terms of desired living arrangement, the estimated coefficients of co-residence and living in the same neighborhood were −0.059 and 0.608, respectively, and the odds ratios were 0.943 and 1.836, respectively; only living in the same neighborhood attained a level of significance. This suggests that respondents who desired to live in the same neighborhood with their sons/daughters/parents have a higher probability of choosing age-friendly housing compared to those who desired to co-reside. Chen [45] indicated that a husband and wife who live together with both their parents is a result of a symmetrical interaction of power between both sexes. Even though traditional norms dictate that the son shoulders the primary responsibility of taking care of his parents. If solely based on living arrangement, females today have a relatively higher position in a society where co-residence has become more prevalent, and modern adult sons/daughters are more inclined to live in the same neighborhood with their elderly parents. Multigenerational-multiunit living arrangements make up one of the three many components of age-friendly housing, therefore, respondents who desired to live in the same neighborhood as their sons/daughters/parents are more inclined to choose age-friendly housing. The empirical results support what we had surmised.

Desired family type had an estimated coefficient of 1.690 and an odds ratio of 5.419 at a 1% level of significance. This suggests that the odds of respondents who desired to be a part of a stem family choosing age-friendly housing was 5.419 times higher than that of those who desired to be a part of a nuclear family. Li and Huang [21] mentioned that the key factors influencing people who desire a stem family are familial interactions between parents and sons/daughters as well as the housing and socioeconomic resources provided by parents. Moreover, sons/daughters were better able to meet their needs when they live in the same neighborhood as their parents compared to co-residing with their parents. According to Wang [19], due to filial norms, people who desired to co-reside with their parents/sons/daughters are less likely to choose housing within the vicinity of their natal home, and are more likely to maintain the “three-generations under one roof” living arrangement. The empirical results support what we had surmised.

House-buying priorities had an estimated coefficient of 0.835 and an odds ratio of 2.304 at a 1% level of significance. This suggests that the odds of respondents who prioritized housing type (age-friendly or ordinary housing) choosing age-friendly housing was 2.304 times higher than that of respondents who prioritized housing location. The empirical results support what we had surmised. The estimated coefficients of high and medium education level were −0.061 and −0.376, respectively, and the odds ratios were 0.941 and 0.686, respectively, which did not attain a level of significance. This suggests that desired housing unit price did not exert any significant differences on the choice probability of age-friendly or ordinary housing. The empirical results do not in support of what we had surmised.

## 6. Conclusion and Recommendations

This questionnaire-based study employed convenience sampling to examine the behaviors of Kaohsiung residents in choosing age-friendly housing, so as to build a choice model regarding age-friendly housing in Kaohsiung City. The empirical results showed that compared to their parents, adult sons/daughters were more accepting of age-friendly housing, a new housing type that offers a better quality of living. Residents who currently live in the city center (which is more convenient and livable) are more willing to purchase age-friendly housing than residents living in other areas. Respondents with a higher family income (who are discreet about their contributions), as well as those with higher social status, are more likely to choose age-friendly housing located in the same neighborhood as other generations of the family. Respondents who are currently or desire to be a part of a stem family are more inclined to choose age-friendly housing located in the same neighborhood as other generations of the family than those who are part of a nuclear family. Respondents who prioritize housing type during house-buying have a higher propensity toward choosing age-friendly housing.

The results of this study can serve as a reference for real-estate investors who seek to invest in age-friendly housing, as well as for consumers who are deliberating between live in age-friendly housing or ordinary housing. In terms of housing attributes, emphasis was placed on age-friendly housing, which provides a better quality and convenience of living, as well as practical services. With regard to multigenerational-multiunit living arrangements and stem families, the emphasis was on filial norms, aging in place, and the exchange theory, that is, parents care for their grandsons/granddaughters, do housework, or manage the household’s socioeconomic resources while their sons/daughters care for them owing to interests, prestige-related, and labor-related factors. Most of the respondents who chose age-friendly housing were from high-income groups and emphasized the grade of building materials, housing quality, and community environment. Therefore, building high-quality age-friendly housing can satisfy the needs of high-income groups. In terms of location, age-friendly housing options should be located in city centers that are convenient, livable, and accessible, so as to reduce time and money spent on transportation. This satisfies the needs of both parents and sons/daughters during different stages of their lives. Hence, when the real estate industry invests in age-friendly housing, such housing should be marketed toward stem families and should be located in city centers where it is more convenient, thus satisfying the needs of consumers.

By covering age-friendly housing, this study distinguishes itself from previous studies, such as those by Szanton et al. [4], Kim et al. [5], Wang et al. [6]. and Scheidt [9] who explored the means of implementing aging in place as well as the living arrangements of elderly people; as well as those of Clark and Onaka [12] and Bonnet [14], who explored house buyers’ intentions to choose ordinary housing. Age-friendly housing is a relatively new housing type, and existing Taiwanese studies that cover age-friendly housing are mostly based on the perspectives of suppliers. These studies concurred that consumers would choose age-friendly housing if such housings offer age-friendly facilities and spatial designs. However, this study indicated that consumers’ choice of age-friendly housing entails various consumer-based dimensions, such as a multigenerational-multiunit living arrangement, the choice behavior of consumers who chose age-friendly housing, and the housing features.

From the perspective of housing market segmentation, prior to its merging into a special municipality, the Kaohsiung metropolitan region consisted of Kaohsiung city and Kaohsiung county, and the city was further divided into north and south Kaohsiung, while the county was divided into the north and south outer peripheries. Each of these areas had different consumer characteristics, which resulted in significant differentiation in the housing sub-markets in the aforementioned areas. In terms of different urban developments in the northern and southern centers of Kaohsiung City as well as the peripheries of Kaohsiung County, most of the land in the northern and southern city centers was densely developed, with mansion apartments and apartments being the prominent housing types, while in the periphery areas, townhouses and apartments were the prominent housing types. Therefore, in order to examine the different consumption patterns in the northern and southern city centers as well as the periphery areas, we suggest that further research should examine Kaoshiung City’s housing sub-market in its metropolitan area. This would shed light on the age-friendly housing needs of consumers from all areas. Age-friendly housing is a relatively new and uncommon housing type in Taiwan and remains unknown to many consumers. Therefore, age-friendly housing has received limited attention from investors and buyers. This study suggests that once age-friendly houses have been built in all the administrative districts of Kaohsiung City, research can be conducted to compare the consumers’ degree of preference before and after living in age-friendly housing units, as well as to analyze the factors affecting their choice of housing and housing needs and the order of their house-buying priorities. In the future, a nested logit model could be employed to analyze the factors and hierarchy of consumers’ needs when choosing age-friendly housing.

## Figures and Tables

**Table 1:** Description of variable settings.

Variable	Measurement Method	Expected Signs
Dependent variable		
Housing type (BCHOICETYPE)	There were two housing types in this study - age-friendly housing and ordinary housing. Respondents who chose age-friendly housing were set as 1 while the others who choose ordinary housing were set as 0.	
Independent variables		
Socioeconomic characteristics		
Generation (GEN)	This study utilized an age of 66 years and above as a reference group, and older adults aged 66 years and above were set as 1 while younger adults aged under 66 years were set as 0.	+
Gender (SEX)	In this study, gender was designed as a dummy variable in which males are set as 1 while females are set as 0.	+
Current living location (CLOC)	Current living location was designed a dummy variable, in which city centers were set as 1 and suburban areas were set as 0.	+
Monthly family income (INCOME)	Monthly family income was divided into low (less than NT$60,000); middle (between NT$60,000 and NT$120,000), and high (over NT$120,000), in which the low income group served as a reference, and two dummy variables (INCOME1 and INCOME2) were designed. In INCOME1, high income buyers were set as 1, while others were set as 0; in INCOME2, middle income buyers were set as 1, while others were set as 0.	+
Education level (EDU)	Education level was divided into low, medium, and high, in which low education level served as the reference group, and two dummy variables (EDUH and EDUM) were designed. In EDUH, high education level was set as 1, while medium and low education level was set as 0. In EDUM, medium education level was set as 1, while high and low education level was set as 0.	+
Current housing attributes		
Current house ownership (COWNSHIP)	In this study, there were three types of current house ownership: consumers’ (or their spouse’s) sole ownership; joint ownership between parents and sons/daughters; and tenancy and others, in which Tenancy and other served as a reference group, and two dummy variables (COWNSHIP1 and COWNSHIP2) were designed. In COWNSHIP1, sole ownership was set as 1, while joint ownership and tenancy and others were set as 0. In COWNSHIP2, joint ownership was set as 1, while sole ownership and tenancy and others were set as 0.	+
Current family type (CTYPE)	Current family type was determined by the number of family members in a family. Family types included nuclear family and stem family, and a dummy variable was designed. A family with one to four current members is a nuclear family while a family with five or more members is a stem family. Consumers with a stem family were set as 1, while consumers with a nuclear family were set as 0.	+
Desired housing attributes		
Desired living location (HLOC)	For the desired living location variable, suburban areas served as the reference group, and a dummy variable (HLOC) was designed, in which city centers were set as 1, and suburban areas were set as 0.	+
Desired living arrangement (HLIVETYPE)	This study divided living arrangement into three types based on distance: parents-sons/daughters co-residence; living in the same neighborhood with each other; and living as far apart as possible. The latter served as the reference group, and two dummy variables (HLIVETYPE1 and HLIVETYPE2) were designed. In the former, parents-sons/daughters co-residence was set as 1, while other living arrangements were set as 0. In the latter, parents and sons/daughters living in the same neighborhood with each other was set as 1, while other living arrangements were set as 0. We surmise that the coefficients of the two desired living arrangement variables have positive values.	+
Desired family type (HFAMTYPE)	Two desired family types were designed, stem families (five members or more) and nuclear families (one to four members), in which nuclear families served as a reference group, and a dummy variable (HFAMTYPE) was designed. Respondents who were part of a stem family were set as 1, while those who were part of a nuclear family were set as 0.	+
House-buying priorities (BCHOICE)	Respondents prioritized two factors - housing type (age-friendly housing and ordinary housing) and housing location (city center or suburban area) when buying a house. Respondents who prioritized housing type were set as 1, while those who prioritized housing location were set as 0.	+
Desired housing unit price (HUP)	There were three types of desired housing unit prices - high (more than NT$180,000); middle (between NT$140,000 and NT$180,000), and lower (NT$140,000), in which lower-priced housing served as a reference group, and two dummy variables (HUPH, HUPM) were designed. In the former, high-priced housing was set as 1, while middle- and lower-priced housing were set as 0. In the latter, middle-priced housing was set as 1, while high- and lower-priced housing were set as 0. As of September 1, 2020, 1$US is equal to 29.75$NT.	−

Note: 1. For the monthly family income variable, the minimum interval was set as less than NT$30,000, as calculated based on Taiwan’s existing minimum wage of NT$23,800. Low- and middle-income groups were classified by means of equal interval classification of the questionnaire items; 2. Education level was classified according to the Ministry of Education Statistics Department’s Classification of Education Levels by Existing School Systems, 5th Revision (January, 2016); 3. Expected housing unit price was classified according to the conventional classification schemes used by the real estate market in Kaohsiung City.

**Table 2:** Descriptive statistics of samples (*n* = 300).

Variable	Frequency	(%)
Generation (GEN)		
Parents (above 66 years of age)	17	5.7
Sons/daughters (below 66 years of age)	283	94.3
Gender (SEX)		
Male	141	47.0
Female	159	53.0
Current living location (CLOC)		
City center	140	46.7
Suburban area	160	53.3
Monthly family income (INCOME)		
Low (below NT$60,000)	124	41.3
Middle (between NT$61,000 and NT$120,000)	124	41.3
High (over NT$121,000)	52	17.4
Education level (EDU)		
Low (elementary school and below)	25	8.3
Medium (senior high school and junior college)	147	49.0
High (university and above)	128	42.7
Current house ownership (COWNSHIP)		
Respondent’s (or their spouse’s) sole ownership	129	43.0
Joint ownership between parents and sons/daughters	111	37.0
Renting and others	60	20.0
Current family type (CTYPE)		
Nuclear family	205	68.3
Stem family	95	31.7
Living in the same neighborhood	83	27.7
Living in the same area	99	33.0
Living as far away as possible	14	4.7
Desired family type (HFAMTYPE)		
Nuclear family	221	73.7
Stem family	79	26.3
Desired housing type (HTYPE)		
Age-friendly housing	151	50.3
Ordinary housing	149	49.7
House-buying priorities (BCHOICE)		
Housing type	123	41.0
Housing location	177	59.0
Desired housing unit price (HUP)		
Lower-priced (less than NT$140,000 per *ping*)	116	38.7
Middle-priced (NT$141,000 to NT$180,000 per *ping*)	106	35.3
High-priced (more than NT$181,000 per *ping*)	78	26.0

**Table 3:** Statistical estimation results (*n* = 300).

Variable	Estimated Coefficient	Standard Error	Wald Statistic	*p*-Value	Odds Ratio
Generation (GEN)	−1.623	0.784	4.293	0.038 **	0.197
Gender (SEX)	−0.150	0.268	0.315	0.575	0.860
Current living location (CLOC)	0.866	0.283	9.350	0.002 ***	2.377
High monthly family income (INCOME1)	1.109	0.435	6.503	0.011 **	3.032
Medium monthly family income (INCOME2)	0.538	0.312	2.978	0.084 *	1.712
High education level (EDUH)	−0.305	0.668	0.209	0.648	0.737
Medium education level (EDUM)	−0.703	0.660	1.134	0.287	0.495
Sole ownership (COWNSHIP1)	0.256	0.384	0.446	0.504	1.292
Joint ownership (COWNSHIP2)	−0.237	0.381	0.388	0.533	0.789
Current family type (CTYPE)	0.589	0.349	2.847	0.092 *	1.802
Desired living location (HLOC)	−0.509	0.314	2.640	0.104	0.601
Co-residence (HLIVETYPE1)	−0.059	0.344	0.029	0.864	0.943
Living in the same neighborhood (HLIVETYPE2)	0.608	0.328	3.436	0.064 *	1.836
Desired family type (HFAMTYPE)	1.690	0.394	18.412	0.001 ***	5.419
House-buying priorities (BCHOICE)	0.835	0.279	8.947	0.003 ***	2.304
High desired housing price (HUPH)	−0.061	0.364	0.028	0.867	0.941
Middle desired housing price (HUP)	−0.376	0.310	1.472	0.225	0.686
LR chi2(17) Prob> chi2Pseudo $R^{2}$	64.100.0010.154				

Note: *, **, and *** indicate *p* < 0.10, 0.05, and 0.01 respectively.

## Appendix A

**Questionnaire items**

1. Please state your birth year:
2. Please select your gender: 1. □ Male 2. □ Female.
3. Please state the administrative district you are currently living in: District.
4. Please state the current owner of your house: 1. □ Myself (or my spouse) 2. □ My parents/son/daughter 3. □ Landlord 4. □ Other.
5. Please state the number of family members that are currently residing with you:
6. Please state the monthly income of your family: 1. □ Less than NT$30,000 2. □ NT$31,000 to NT$60,000 3. □ NT$61,000 to NT$90,000 4. □ NT$91,000 to NT$120,000 5. □ NT$121,000 to NT$150,000 6. □ More than NT$151,000.
7. Please state your highest level of education attained: 1. □ Lower than elementary school and below 2. □ Elementary school 3. □ Junior high school 4. □ Senior (vocational) high school 5. □ Associate’s degree 6. □ Bachelor’s degree (includes two-year and four-year technical programs) 7. □ Master’s degree 8. □ Doctoral degree.
8. Please state the preferred location of your purchased house if you decide to move out: 1. □ The city center (Sinsing, Cianjin, Lingya, Sanmin, Gushan, Tsoying, and Fongshan districts) 2. □ The suburban area (Yancheng, Cianjhen, Siaogang, Nanzih, Ciaotou, Renwu, Niaosong, and Daliao districts).
9. Please state your desired living arrangement if you decide to move out: 1. □ Living in the same house with parents/son/daughter 2. □ Living in the same neighborhood with parents/son/daughter 3. □ Living in the same area with parents/son/daughter 4. □ Living as far away from parents/son/daughter as possible.
10. Please state the number of family members that you desire to reside with in the future if you decide to move out:
11. Please state your house-buying priority if you decide to move out: □ Housing type (age friendly housing/ordinary housing) 2. □ Housing location (city center/suburban area).
12. Please state your desired housing type if you decide to move out: 1. □ Age-friendly housing 2. □ Ordinary housing.
13. Please state your desired room type if you decide to move out: 1. □ Suite 2. □ 1 bedroom 3. □ 2 bedrooms 4. □ 3 bedrooms 5. □ 4 bedrooms 6. □ 5 bedrooms or more.
14. Please state your desired housing floor area if you decide to move out: 1. □ Less than 20 *ping* 2. □ 21 to 30 *ping* 3. □ 31 to 40 *ping* 4. □ 41 to 50 *ping* 5. □ 51 to 60 *ping* 6. □ More than 61 *ping*.
15. Please state your desired housing total price if you decide to move out: 1. □ Less than NT$3 million 2. □ NT$3.01 to NT$5 million 3. □ NT$5.01 to NT$7 million 4. □ NT$7.01 to NT$9 million 5. □ NT$9.01 to NT$11 million 6. □ NT$11.01 to NT$13 million 7. □ NT$13.01 to NT$15 million 8. □ More than NT$15.01 million.
16. Please state your desired housing unit price (NT$ per *ping*) if you decide to move out: 1. □ Below $140,000 2. □ NT%141,000 to NT$150,000 3. □ NT$151,000 to NT$160,000 4. □ NT$161,000 to NT$170,000 5. □ NT$171,000 to NT$180,000 6. □ NT$181,000 to NT$190,000 7. □ NT$191,000 to NT$200,000 8. □ NT$ NT$201,000 to NT$210,000 9. □ NT$211,000 to NT$220,000 10. □ NT$221,000 to NT$230,000 11. □ NT$231,000 to NT$240,000 12. □ More than NT$241,000.
17. Please state your acceptable housing price range if you decide to move out: 1. □ Equivalent to the quoted price 2. □ by 1% to 5% higher than the quoted price 3. □ 6% to 10% higher than the quoted price 4. □ 11% to 15% higher than the quoted price 5. □ 16% to 20% higher than the quoted price.
18. Please state the acceptable management fee range of your purchased house after you have moved out: 1. □ Less than NT$60 per *ping* 2. □ NT$61 to NT$70 per *ping* 3. □ NT$71 to NT$80 per *ping* 4. □ NT$81 to NT$90 per *ping* 5. □ More than NT$91 per *ping.*
19. Please state your minimum desired loan-to-value ratio if you decide to move out: 1. □ No loan required 2. □ Less than 30% 3. □ 31% to 40% 4. □ 41% to 50% 5. □ 51% to 60% 6. □ 61% to 70% 7. □ 71% to 80% 8. □ 81% to 90% 9. □ 91% to 100%.
20. Please check the factors that influence your choice of age-friendly housing(multiple choice): 1. □ Availability of age-friendly public infrastructure 2. □ Multigenerational-multiunit living arrangements 3. □ Lifetime home designs 4. □ Flexible space planning 5. □ Safety information system 6. □ Remote care services 7. □ Holistic spatial mechanisms. ~ Applicable for those who answered “Age-friendly housing” in Question 4 only; please leave this question blank if your answered “Ordinary housing.”
21. Please check the factors that influence your choice against age-friendly housing (multiple choice): 1. □ Availability of age-friendly public infrastructure 2. □ Multigenerational-multiunit living arrangements 3. □ Lifetime home designs 4. □ Flexible space planning 5. □ Safety information system 6. □ Remote care services 7. □ Holistic spatial mechanisms. ~ Applicable for those who answered “Ordinary housing” in Question 4 only; please leave this question blank if your answered “Age-friendly housing.”

## References

[1] Forsyth A., Molinsky J., Kan H.Y. (2019). Improving housing and neighborhoods for the vulnerable, older people, small households, urban design, and planning. Urban Des. Int..

[2] Hwang Y.R. (2006). Feasibility of Aging in Place and Foundation of Life-time Housing Development. Taiwan Geriatr. Gerontol..

[3] Chen C.H. (2006). The Concept of Total Planning for Senior Housing. Taiwan Geriatr. Gerontol..

[4] Szanton S.L., Leff B., Wolff J.L., Roberts L., Gitlin L.N. (2016). Home-based care program reduces disability and promotes aging in place. Health Affairs.

[5] Kim K.I., Gollamudi S.S., Steinhubl S. (2017). Digital technology to enable aging in plac. Exp. Gerontol..

[6] Wang S., Bolling K., Mao W., Reichstadt J., Jeste D., Kim H.C., Nebeker C. (2019). Technology to Support Aging in Place: Older adults’ perspectives. Healthcare.

[7] Ahn M., Kwon H.J., Kang J. (2020). Supporting aging-in-place well: Findings from a cluster analysis of the reasons for aging-in-place and perceptions of well-being. J. Appl. Gerontol..

[8] Sanguinetti A. (2015). Diversifying cohousing: The retrofit model. J. Archit. Plan. Res..

[9] Scheidt R.J. (2017). Coming of age in aging America. Gerontologist.

[10] Xie X.L., Liu C.W., Zhu Y.F. (2020). Study on the Item Development and Distribution Model of an Age-Friendly Retirement Community. Hous. Sci..

[11] Chen M.Y. (2019). Building a Friendly and Smart Living Space Suitable for All Ages. Consum. Rep. Taiwan.

[12] Clark W.A., Onaka J.C. (1985). An empirical test of joint model of residential mobility and housing choice. Environ. Plan..

[13] Bhat C.R. (2015). A comprehensive dwelling unit choice model accommodating psychological constructs within a search strategy for consideration set formation. Transp. Res. Part B.

[14] Bonnet O. (2018). Individual Housing Choices and Aggregate Housing Prices: Discrete Choice Models Revisited with Matching Models. Ph.D. Thesis.

[15] Coulter R. (2018). Parental background and housing outcomes in young adulthood. Hous. Stud..

[16] Hsieh C.L., Tsai R.U. (2014). Urban Development and Quality of Life: A Study on Housing Choices of the Elderly and Younger Generations. J. Reg. Soc. Dev. Res..

[17] Wang C.M. (2015). Cross-Generation in the Same Neighborhood 1 + 1 Residential Living Pattern Study—Blue Ocean Strategy of Yungching Real Estate Inc.. Master’s Thesis.

[18] Yi C., Lin J. (2009). Types of relations between adult children and elderly parents in Taiwan: Mechanisms accounting for various relational types. J. Comp. Fam. Stud..

[19] Wang H.-C. (2013). The Effect of Co-Residence Preference and Filial Norms on Intergenerational Living Arrangement.

[20] Huang Y.T. Intergenerational Relationships and Adult Children Support to Ageing Parents in Taiwan. Proceedings of the 10th Annual Conference on Development Studies—Taiwan Experience 2.0: Local and Global Development Studies and Practices.

[21] Shih Y.P. (2019). Parenting in Three-generation Taiwanese families: The dynamics of collaboration and conflicts. Contemp. Perspect. Fam. Res..

[22] Huang C.H. (2017). A Study on the Housing Requirement on All-Age Housing Community: A Case Study in Tainan City. Master’s Thesis.

[23] Naess P., Peter S., Stefansdottir H., Strand A. (2018). Causality, not just correlation: Residential location, transport rationales and travel behavior across metropolitan contexts. J. Transp. Geogr..

[24] Hsiang C.K. (2015). A Study of Affecting Factors on the Decision making of House Purchasing-A Case Study in Kaohsiung. Master’s Thesis.

[25] Tseng L.Y., Chign C.H., Chen S.M. (2006). An Analysis on the Living Arrangement Choices of the Elderly: A Discussion on Intergenerational Relationships. J. Hous. Stud..

[26] Kuo H.P. (2010). Effects of Filial Piety on Living Arrangement between Baby Boomers and Generation-X. Master’s Thesis.

[27] Lin Y.H., Peng C.W. (2019). Married young adults living with parents-an analysis of regional differences. Int. Real. Estate Rev..

[28] Chang G.L., Chang C.O. (2010). Transitions in Living Arrangements and Living Preferences among Elderly: An Analysis from Family Values and Exchange Theory. J. Popul. Stud..

[29] Lin C.C. (1993). A Review and Outlook of Methods for Assessing the Requirements in Adult Education in Taiwan. Adult Educ. Bimon..

[30] Chien W.Y., Yi C.C. (2001). The Dynamic Development of Taiwanese Families: Structural Fission and Expansion. J. Popul. Stud..

[31] Chao T.Y. (2004). City Living and Empty Nesters—Attitudes to Mixed-Use Areas. Ph.D. Thesis.

[32] Lee H.Y. (2017). The Research on the Influences of Population Structure Change Scenarios on Housing Purchase Behavior Preferences: A Study of Taipei City. Master’s Thesis.

[33] Hu C.P. (2013). Model for Housing Duality and Household Movement Decision. J. Archit. Plan..

[34] Lein C.Y. (2003). Fuzzy Linguistic Approach and Discrete Choice Theory for Building Choice Behavior Model in Household Purchase. Ph.D. Thesis.

[35] Tefera B.P.D. (2019). Factors Affecting Real Estate Customers Choice of Residential Houses; Evidence from Some Selected Real Estate Ccompanies in Ethiopia. Master’s Thesis.

[36] Li W.D., Hung C.Y. (2019). Parental support and living arrangements among young adults in Taiwan. J. Hous. Built Environ..

[37] Chang T.-C. (2013). Gender Selection in Destination Choices of Labor Migration. J. Hous. Stud..

[38] Liu K.S., Liao Y.T., Hsueh S.L. (2017). Implementing smart green building architecture to residential project based on Kaohsiung, Taiwan. Appl. Ecol. Environ. Res..

[39] Hsu K.C. (2019). Effect of distinct land use patterns on quality of life in urban settings. J. Urban Plan. Dev..

[40] Chang C., Chen S., Somerville T. (2003). Economic and social status in household decision-making: Evidence relating to extended family mobility. Urban Stud..

[41] Chen C.L., Lin C.C. (1998). Wealth Effect, Income Effect, and Housing Demand. J. Hous. Stud..

[42] Yi C.C., Chang C.F. (2019). Family and gender in Taiwan. East Asian Gend. Man..

[43] Chen C.Y. (2018). The Effects of Housing Tenure on the Subjective Social Status and Self-Rated Health Status of the Elderly. J. Hous. Stud..

[44] Kim T., Horner M.W. (2003). Exploring spatial effects on urban housing duration. Environ. Plan. A.

[45] Chen C.L. (2005). Parent-child Living Arrangement under Intra-& Inter-Household Members’ Power Interaction. J. Hous. Stud..

